# Major Bleeding Events Are Stronger Predictors of Long-Term Mortality Than Coronary Events in Secondary Prevention Therapy for Ischaemic Heart Disease

**DOI:** 10.1155/2020/9303750

**Published:** 2020-11-30

**Authors:** Shigetaka Kageyama, Koichiro Murata, Ryuzo Nawada, Tomoya Onodera, Yuichiro Maekawa

**Affiliations:** ^1^Department of Cardiology, Shizuoka City Shizuoka Hospital, Shizuoka, Japan; ^2^Internal Medicine III, Hamamatsu University School of Medicine, Shizuoka, Japan

## Abstract

**Background:**

Secondary prevention of ischaemic heart disease (IHD) is an important aspect of healthcare. To improve the prognosis of and control risk factors for IHD patients, we created a unique referral system called the Shizuoka IHD patient registry.

**Methods:**

From 2009 to 2013, we enrolled 1240 patients; they participated in follow-up until 2018. The risk factor target values were as follows: low-density-lipoprotein cholesterol, <100 mg/dl; glycated haemoglobin of diabetes patients, <7%; systolic blood pressure, <130 mmHg; and diastolic blood pressure, <80 mmHg (mean follow-up interval, 2001 ± 794 days). The cumulative incidence rates were 10.8% for all-cause death (cardiac death, 1.5%), 15.7% for coronary events, and 2.6% for major bleeding. Patients were separated into the major bleeding group (*n* = 32), coronary event group (*n* = 195), and event-free group (*n* = 1013) without overlapping.

**Results:**

We observed significant differences in age, rate antithrombotic drug use, and mortality. A Kaplan–Meier analysis of all-cause death showed significant differences between the event-free and major bleeding groups (*P*=0.002) and between the coronary event and major bleeding groups (*P*=0.026); there was no significant difference between the event-free and coronary event groups.

**Conclusion:**

Major bleeding events were stronger predictors of long-term mortality than coronary events during the long-term follow-up of stable IHD.

## 1. Introduction

There are established evidence and guidelines for patients with ischaemic heart disease (IHD) to achieve secondary prevention. Nevertheless, the long-term prognosis of these patients is unknown, and the current evidence is based on trials mainly performed at specialised centres with experienced cardiologists [1–3]. However, most patients whose conditions are stabilised with optimal medical therapy, percutaneous coronary intervention (PCI), and coronary artery bypass grafting are treated by general practitioners. General practitioners usually evaluate the conditions of their patients using simple examinations and prescribe medications accordingly.

Secondary prevention of IHD is an unobtrusive yet important aspect of healthcare [4, 5]. Because the long-term prognosis of IHD patients is unknown, and because Shizuoka city has fewer cardiologists than is usual according to the Japanese standards, we decided to establish a unique referral system to connect the hospital and outpatient clinics.

This study aimed to introduce our unique audit referral system and present the long-term prognosis for IHD patients undergoing optimal secondary prevention therapy for a decade.

## 2. Methods

### 2.1. Study Design, Establishment of the Referral System, and Patient Recruitment

The Shizuoka Ischaemic Heart Disease registry was established in 2009 to connect cardiologists in Shizuoka City Hospital with more than 200 general practitioners in Shizuoka City. The registry is for patients who undergo interventions and those who need only optimal medical therapy.

**Figure 1:**
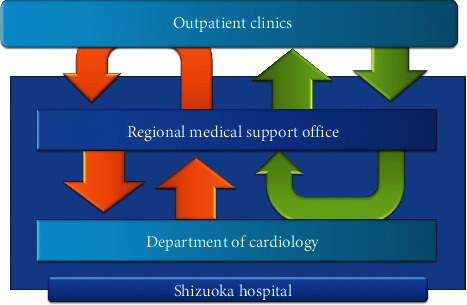
Flowchart of the ischaemic heart disease referral system.

To audit the treatment provided by general practitioners, we adopted a circulation-type cooperative form. General practitioners were required to present registered patients at least once per year even if no cardiac events occurred. They were also asked to examine electrocardiographs every 3 months and order blood sampling tests for risk factors every 6 months. We created a simple form for referral to determine whether the patients' risk factors were well controlled (Figure 1). The first meeting occurred in May 2009; since then, the registration process has been ongoing. From May 2009 to December 2013, 1286 patients were enrolled in the registry. Patients eligible for inclusion in this registry were those with IHD who were referred by the hospital to their general practitioners in a relatively stable condition. There were no exclusion criteria, with or without intervention, and actual inclusion was at the discretion of the hospital's cardiologists.

Data from yearly follow-up visits, including vital signs and physical examination findings, electrocardiograms, chest X-rays, and laboratory tests, were collected by the investigators upon hospital visits. Additional follow-up information, including vital signs, mortality, additional hospitalisations, and the status of antiplatelet therapy, were collected by contacting the patients, their relatives, or referring physicians via a questionnaire or telephone calls (patients only). Data of patients who were lost to follow-up were removed from the follow-up dataset on the last day of the study.

The study was conducted in accordance with the Declaration of Helsinki and was approved by an institutional review board. Oral informed consent was obtained from the enrolled patients.

In 2015, the registry was expanded to a multicentre study involving three core hospitals in Shizuoka city. However, for this study, we used the data extracted from only our institute.

We followed up a total of 1240 patients (follow-up rate, 96.4%) who were divided into three groups based on major bleeding events, coronary events, and absence of any events. The median follow-up interval was 2106 days (interquartile range, 1475–2596 days).

### 2.2. End Points and Definitions

Death was defined as cardiac in origin unless obvious noncardiac causes could be identified. Any death during the index hospitalisation was regarded as cardiac death. Angina pectoris (AP) and myocardial infarction (MI) were evaluated according to the latest guidelines of the Japanese Circulation Society based on the universal definition [6]. Coronary events were defined as a combination of cardiac death, MI, and AP that required hospitalisation including revascularisation therapy. Major adverse cardiac and cerebrovascular events (MACCE) were defined as a combination of coronary events, ventricular arrhythmia, congestive heart failure (CHF) requiring hospitalisation, and cerebral infarction.

Bleeding events were recorded when the patient required discontinuation of antiplatelet drugs, hospitalisation, or blood transfusions. Major and minor bleeding were defined by the thrombolysis in MI bleeding criteria [7].

### 2.3. Risk Factor Management

The recommended antiplatelet regimen after PCI was aspirin (100 mg daily) indefinitely and thienopyridine (200 mg ticlopidine or 75 mg clopidogrel daily or 3.75 mg prasugrel daily) for at least 1 year. The duration of dual antiplatelet therapy (DAPT) was based on the discretion of attending cardiologist at the hospital. We set a target value that adheres to the latest Japanese guidelines for the secondary prevention of IHD [8]. Statins were recommended or the existing dosage was increased when the low-density-lipoprotein cholesterol (LDL-C) concentration was more than 100 mg/dL. Improvements in diet and the addition of oral hypoglycaemic drugs were recommended if the glycated haemoglobin (HbA1c) level was more than 7%. Antihypertensive drugs were recommended if the systolic blood pressure (SBP) was more than 130 mmHg and/or the diastolic blood pressure (DBP) was 80 mmHg at home.

### 2.4. Statistical Analysis

Categorical variables were expressed as numbers and percentages. Continuous variables were expressed as means ± standard deviation unless otherwise indicated. The characteristics of patients across two groups were compared using *t*-tests for continuous variables and chi-squared tests for categorical variables. A multivariate analysis was performed using binary logistic regression when the univariate analysis showed statistically significant differences. To compare all three groups, a nonparametric analysis was performed because equal variance could not be assumed. Cumulative incidence rates were calculated using the Kaplan–Meier method.

All statistical analyses were performed using SPSS Statistics 21 (IBM Corporation, Chicago, IL). All reported *P*-values were two-sided, and *P* < 0.05 was considered statistically significant. Significant predictors of clinical events were presented with odds ratios (OR) and 95% confidence intervals (CI).

## 3. Results

### 3.1. Baseline Characteristics

Among the 1286 patients who were registered from 2009 to 2013, 925 attended consultations at the hospital based on their predetermined medical examinations. Data regarding clinical events were available for 315 nonconsulted patients from the primary care physician or through telephone interviews. Forty-six patients were lost to follow-up. Therefore, we followed up 1240 patients, all of whom survived with a confirmed prognosis until 2018. The mean age was 77.3 ± 10.6 years and 76.3% were men. Patients with MI accounted for 39.6%, and 33% had diabetes at registration. The DAPT rate was 52.3%. The statin prescription rate was 75.7%. Patients were prescribed the following: mineralocorticoid receptor antagonists, 60.9%; beta-blockers, 24.1%; and calcium channel blockers (CCB), 55%. Risk factor control at registration showed the following results: SBP, 126.6 ± 17.5 mmHg; DBP, 69.5 ± 12.6 mmHg; LDL-C, 93 ± 28.5 mg/dL; and HbA1c in diabetes patients, 6.7 ± 1.0%. Baseline characteristics of the patients are presented in Table 1.

### 3.2. Changes in Morbidity and Medication

The proportion of patients diagnosed with diabetes at the latest follow-up increased to 36.1%, whereas that of patients who required antihypertensive drugs and statins increased to the 90% range. The DAPT rate decreased to 52.3%. Oral anticoagulation (OAC) therapy was administered to 11.7% of patients. The results of changes in morbidity and medications are shown in Table S1.

### 3.3. Changes in Risk Factor Control

The average LDL-C level significantly decreased from baseline to follow-up (93 ± 28.5 to 88.0 ± 21.3 mg/dL; *P* < 0.001). HbA1c control in diabetes patients significantly worsened (6.7 ± 1.0 to 7.0 ± 1.1%; *P* < 0.001). Moreover, there was a significant increase in both SBP (126.6 ± 17.5 to 133.7 ± 16.5 mmHg; *P* < 0.001) and DBP (69.5 ± 12.6 to 75.0 ± 11.8 mmHg; *P* < 0.001).

### 3.4. Incidences of Clinical Events

Over the course of the 5- to 10-year follow-up period, the cumulative incidence of all-cause death was 10.8% (*n* = 135). Cardiac death occurred in 1.5% of patients (*n* = 18) and MACCE in 19.8% (*n* = 246). The proportions of patients according to the classification of MACCE, except cardiac death, were as follows: MI, 1.6% (*n* = 20); AP with hospitalisation, 4.6% (*n* = 57); any lesion revascularisation, 4.5% (*n* = 56); target lesion failure (TLF), 12.9% (*n* = 160); CHF, 3.0% (*n* = 37); ventricular arrhythmia, 1.2% (*n* = 15); and cerebral infarction, 1.1% (*n* = 13) (Table 2). We defined a coronary event as the composite end point of cardiac death, MI, AP hospitalisation, any lesion revascularisation, and TLF. The cumulative incidence of coronary events was 15.7% (*n* = 195).

Major bleeding events occurred in 3.1% of patients (*n* = 32), intracranial haemorrhage occurred in 0.9% (*n* = 11), gastrointestinal bleeding occurred in 2.0% (*n* = 19), and other complications occurred in 0.2% (*n* = 2) during follow-up (Table 2). There was no overlap of patients between the coronary event group and major bleeding group.

### 3.5. Causes of Death

**Figure 2:**
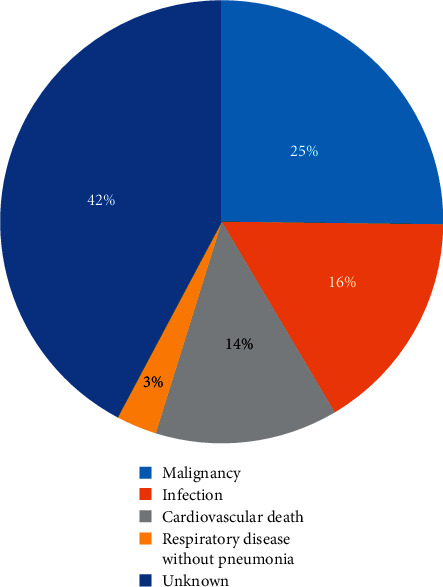
Causes of death for patients with ischaemic heart disease.

The most common cause of death was malignancy (25.1%; *n* = 34), followed by infection, including pneumonia and sepsis (16.3%; *n* = 22), cardiac death (13.3%; *n* = 18), and major bleeding (8.1%; *n* = 11). Despite every effort, the cause of death could not be identified for 28.9% of patients. Classifications of all causes of death are shown in Figure 2.

### 3.6. Predictors of All-Cause Death

Age at baseline was significantly older in the all-cause death group than in the event-free group (82.9 ± 8.1 vs. 76.6 ± 10.6 years; *P* < 0.001). The incidence rates of MI (4.5% vs. 1.3%; *P*=0.005), ventricular arrhythmia (3.1% vs. 1.0%; *P*=0.042), and intracranial haemorrhage (5.3% vs. 0.4%; *P* < 0.001) were higher in the all-cause death group than in the event-free group.

The following factors that showed significant differences between groups were significant predictors in the multivariate analysis: age (OR, 1.07; 95% CI, 1.05–1.09), MI (OR, 5.76; 95% CI, 1.81–18.34), ventricular arrhythmia (OR, 4.14; 95% CI, 1.19–14.48), and intracranial haemorrhage (OR, 13.19; 95% CI, 3.58–48.65) (Table 3).

The Kaplan–Meier analysis for categorical variables indicated that all significant predictors showed significant differences in the log-rank test (MI, *P* < 0.001; ventricular arrhythmia, *P*=0.011; and intracranial haemorrhage, *P* < 0.001) (Figures S1–S3).

### 3.7. Prognosis of the Coronary Event, Major Bleeding, and Event-Free Groups

**Figure 3:**
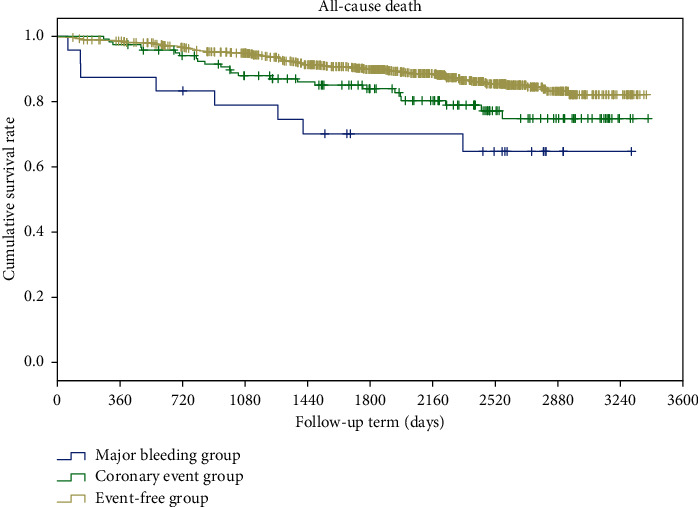
Kaplan–Meier analysis of survival rates of the major bleeding group, coronary event group, and event-free group.

There was no overlap of patients across the three groups. There were 32 patients in the major bleeding group, 195 in the coronary event group, and 1013 in the event-free group. Because we could not expect equal dispersion among the three groups, a nonparametric test (Kruskal–Wallis) was used to evaluate the group differences. The analysis showed significant differences among these groups in age (*P*=0.026), antithrombotic drug use (*P* < 0.001), and mortality (*P*=0.017). The Kaplan–Meier analysis of all-cause death showed a significant difference between the event-free group and the major bleeding group (log-rank *P*=0.002) and between the coronary event group and the major bleeding group (*P*=0.026); however, no significant difference existed between the event-free group and the coronary event group (Table 4, Figure 3).

### 3.8. Variations in Antithrombotic Drug Use and Relations with All-Cause Death and Major Bleeding

We evaluated the variations in antithrombotic drug use according to the presence or absence of all-cause death or major bleeding events using both the baseline and current medications.

**Figure 4:**
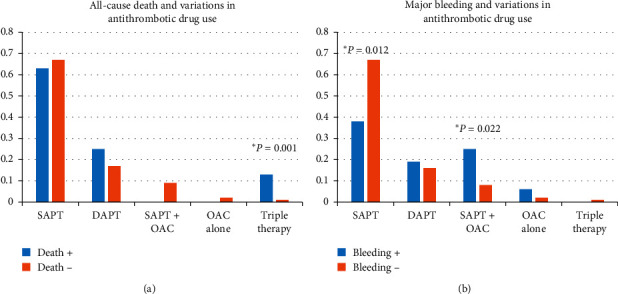
Variations in current antithrombotic drug use with or without all-cause death or major bleeding. DAPT: dual antiplatelet therapy; SAPT: single antiplatelet therapy; and OAC: oral anticoagulation.

We could not find any significant difference at the baseline. However, with regard to the current medication, the all-cause death group had a significantly higher rate of triple antithrombotic therapy than the event-free group (12.5% vs. 8.7%, *P*=0.001). The major bleeding group had a significantly lower rate of single antiplatelet therapy (SAPT) than the event-free group (37.5% vs. 67.3%, *P*=0.012) despite having a significantly higher rate of combined SAPT and OAC use than the event-free group (25% vs. 8.5%, *P*=0.022) (Figure 4).

## 4. Discussion

To the best of our knowledge, this is the first study to reveal the long-term prognosis of IHD patients in the real world using a unique audit referral system. Using this method, general practitioners could follow up these patients in the same manner as cardiologists. Our results suggest that we could audit at least four times as many patients using this approach than with the conventional outpatient system at our hospital. The number of physicians per 1000 people in the Shizuoka Prefecture is 1.9, which is the fourth smallest ratio in Japan. Therefore, especially in these circumstances, the referral system may contribute significantly to patient healthcare.

The development of interventional cardiology has been remarkable. Despite the advent of new devices and methods, objective end point rates have not notably improved for several years. Nevertheless, in the meta-analysis of more versus less intensive statin therapy, the all-cause mortality was reduced by 10% per 1.0 mmol/L decrease in LDL, largely reflecting significant reduction in deaths due to coronary heart diseases and other cardiac causes [9]. Although secondary prevention of IHD is a modest and steady task, its impact on the prognosis of a patient is as important as that of a treatment breakthrough.

Recent outcomes of secondary prevention after an IHD event with conventional optimal medical therapy were reported by several international, multicentre, prospective, randomised studies. In the FOURIER study, the cumulative incidence of cardiac death in the control group was 1.7% and that of MI was 4.6% within a 2.2-year follow-up period [10]. After 6-years of follow-up of patients with acute coronary syndrome in the IMPROVE-IT study, the overall cardiovascular death rate was 5.3% and MI occurred in 10.8% [11]. According to the CREDO-Kyoto AMI registry (2005–2007), the cumulative 5-year incidence of all-cause death in the current study population was 20.4% (cardiac death, 12.2%; noncardiac death, 9.4%). Noncardiac death accounted for nearly two-thirds of all-cause deaths beyond 6 months [12]. However, our referral system improved the patients' prognoses to the same extent as that of conventional optimal medication treatment groups in recent large-scale clinical trials conducted by cardiologists. An observational study based on data from 2199 Copenhagen patients with stable coronary heart disease showed a 6-year mortality rate of 20.1% [13].

We compared the coronary event, major bleeding, and event-free groups. Unexpectedly, neither the nonparametric analysis nor the Kaplan–Meier analysis showed significant differences between the coronary event group and the event-free group. A subanalysis of mortality with or without MI before and after registration also showed no significant difference. According to the KiCS PCI registry (2009–2011), the incidence of acute coronary syndrome (ACS) was 6.4%, the unplanned revascularisation rate was 5.9%, and the all-cause mortality rate was 3.9%. In the propensity matched cohorts, patients with a subsequent admission for ACS had an increased risk of mortality (hazard ratio, 4.73; *P*=0.015), whereas those with unplanned revascularisation did not have a significantly higher risk [14].

In the current study, major bleeding events were more important factors than coronary events. Intracranial haemorrhage directly caused death in many cases. Other major bleeding events always led to the cessation of antiplatelet therapy, either temporarily or permanently. Discontinuation of antiplatelet therapy possibly caused new cardiovascular events, including stent thrombosis. Secondary events worsened the survival rate of the major bleeding group. Coincidentally, the major bleeding group and coronary event group had no overlap of patients in our study. Mortality was estimated to occur more frequently in the major bleeding group than in the coronary event group. The DAPT score is a novel decision tool that was recently developed to identify those who are more likely to benefit from long-term therapy among patients eligible for long-term DAPT [15]. In the present study, DAPT did not contribute to major bleeding or mortality, although the use of a combination of SAPT and OAC therapy was significantly higher in the major bleeding group, use of SAPT and OAC was not associated with all-cause death. De Rosa et al. conducted a meta-analysis involving four randomised controlled trials with a total of 8654 patients to evaluate the safety of multi-antithrombotic therapy in patients with atrial fibrillation undergoing PCI. The dual antithrombotic therapy with direct OAC arm (14.4%) had a significantly lower rate of the primary safety endpoint than the triple antithrombotic therapy with warfarin arm (23%) (*P* < 0.001). In addition, no significant difference was found in the incidence of stroke between the treatment arms (*P*=0.23). Similarly, no significant difference was observed in the incidence of stent thrombosis between the treatment arms (*P*=0.08) [16]. Indeed, in the current study, we found that the all-cause death group had a significantly higher rate of triple antithrombotic therapy than the event-free group. The older the patient during follow-up, the higher the risk of morbidity due to atrial fibrillation. The latest European Society of Cardiology guidelines indicated that OAC therapy alone is an option for patients with chronic IHD [17]. Hence, OAC therapy may be the gold standard for controlling chronic IHD in the elderly Asian population.

There were some limitations to this study. One major limitation involved patient selection. Hospital cardiologists selected and registered patients who were asked to comply with the referral system. The cardiologists tended to follow up patients who were at “high risk” with low ejection fraction (EF); patients with recurrent cardiovascular disease, including those who were postcardiovascular surgery and/or device implantation, and patients with poor adherence and/or poor risk factor control, especially those requiring follow-up for endocrine and metabolic conditions. This study was a prospective, nonrandomised trial. Generally, echocardiographic findings, especially EF, are significant predictors of the prognosis for IHD patients. We did not have adequate resources to perform yearly echocardiography examinations for every referred patient. Our referral system only recommends better medication to the practitioners; there is no enforcement mechanism. The preference of treatment varies based on the practitioners and cardiologists, and treatment strategies are thought to be inhomogeneous. Nevertheless, this diversity is the essence of this research.

We continue to enrol and follow up patients in the referral system. Propensity score matching may be considered for registered patients and patients controlled by hospital visits as a method of evaluating and confirming the usefulness of this system.

## 5. Conclusions

This unique referral system for secondary prevention of cardiovascular disease followed by general practitioners in Shizuoka city showed a good risk factor control and a moderate event rate compared to the previous major studies conducted by cardiologists only. In this study, MI, ventricular arrhythmia, and intracranial haemorrhage after registration were significant predictors of all-cause death. Furthermore, major bleeding events were stronger predictors than coronary events.

## Figures and Tables

**Table 1:** Baseline characteristics of the patients with ischaemic heart disease.

Patient characteristics		*N* = 1240
****Age, years		77.3 ± 10.6
****Male, *n* (%)		946 (76.3%)
****BMI, kg/m^2^		23.4 ± 3.2
****Myocardial infarction, *n* (%)		491 (39.6%)
****Angina pectoris, *n* (%)		709 (57.2%)
****Vasospastic angina, *n* (%)		40 (3.2%)
****Atrial fibrillation, *n* (%)		93 (7.5%)

*Risk factors*		
** **Hypertension, *n* (%)		1085 (87.5%)
** **Diabetes mellitus, *n* (%)		413 (33.3%)
** **Dyslipidaemia, *n* (%)		935 (75.4%)
** **BP, mmHg	Systolic	126.6 ± 17.5
	Diastolic	69.5 ± 12.6
** **HbA1c in DM patients, %		6.7 ± 1.0
** **LDL-C, mg/dL		93.0 ± 28.5

*Medication*		
** **Antiplatelet therapy, *n* (%)		1156 (93.2%)
** **Aspirin		89.7%
** **Thienopyridine		56.6%
** **Cilostazol		1.5%
** **Single		40.4%
** **Dual		52.3%
** **Triple		0.5%
** **Oral anticoagulation therapy, *n* (%)		103 (8.3%)
** **Statin, *n* (%)		939 (75.7%)
** **ACEI and ARB, *n* (%)		755 (60.9%)
** **Beta-blocker, *n* (%)		299 (24.1%)
** **CCB, *n* (%)		682 (55.0%)

*Intervention*		
** **CABG, *n* (%)		161 (13.0%)
** **PCI, *n* (%)		880 (71.0%)

Values are presented as mean ± standard deviation unless otherwise indicated. BMI: body mass index; BP: blood pressure; HbA1c: glycated haemoglobin; LDL-C: low-density lipoprotein cholesterol; ACEI: angiotensin-converting enzyme inhibitor; ARB: angiotensin receptor blocker; CCB: calcium channel blocker; CABG: coronary artery bypass grafting; and PCI: percutaneous coronary intervention.

**Table 2:** Clinical events during follow-up of patients with ischaemic heart disease.

Clinical events	Cumulative incidence, *n* (%)
****All-cause death	135 (10.9%)
****Cardiac death	18 (1.5%)
****MI	20 (1.6%)
****AP	57 (4.6%)
****Any lesion revascularisation	56 (4.5%)
****CHF	37 (3.0%)
****VT/VF	15 (1.2%)
****Ischaemic stroke	13 (1.1%)
****MACCE	246 (19.8%)
****Coronary event	195 (15.7%)

Bleeding events	Cumulative incidence, *n* (%)
** **All-cause bleeding	32 (2.6%)
** **Intracranial haemorrhage	11 (0.9%)
** **Gastrointestinal bleeding	19 (1.5%)
** **Other major bleeding events	2 (0.2%)

Values are presented as mean ± standard deviation, unless otherwise indicated. MI: myocardial infarction; AP: angina pectoris; CHF: congestive heart failure; VT/VF: ventricular tachycardia/fibrillation; and MACCE: major adverse cardiac and cerebrovascular events.

**Table 3:** Risk factors for all-cause mortality in patients with ischaemic heart disease.

Factor	With event(s)	Without event(s)	Univariate analysis *P*-value	Multivariate analysis		
				OR	95% CI	P-value
Baseline						
** **Age	82.9 ± 8.1	76.6 ± 10.6	<0.001	1.07	1.05–1.09	<0.001

Events after registration						
** **MI	4.5%	1.3%	0.005	5.76	1.81–18.34	0.003
** **VT/VF	3.1%	1.0%	0.042	4.14	1.19–14.48	0.026
** **Intracranial haemorrhage	5.3%	0.4%	<0.001	13.19	3.58–48.65	<0.001

Values are presented as means ± standard deviation unless otherwise indicated. MI: myocardial infarction; VT/VF: ventricular tachycardia/fibrillation; OR: odds ratio; and CI: confidence interval.

**Table 4:** Nonparametric test of the major bleeding, coronary event, and event-free groups.

Factor	Major bleeding group (*n* = 32)	Coronary event group (*n* = 195)	Event-free group (*n* = 1013)	*P*-value
Age, years	81.6 ± 10.3	77.3 ± 10.3	77.1 ± 10.5	0.026
DAPT at latest follow-up	18.8%	33.3%	13.8%	<0.001
SAPT + OAC at latest follow-up	25.0%	14.4%	7.6%	<0.001
Mortality rate	25.0%	12.4%	9.9%	0.017

Values are presented as means ± standard deviation unless otherwise indicated. DAPT: dual antiplatelet therapy; SAPT: single antiplatelet therapy; OAC, oral anticoagulation.

## Data Availability

The data used to support the findings of this study are available from the corresponding author upon request.

## Supplementary Materials

Supplementary Materials
*Table S1*. Current morbidity, risk factor control, and medication for patients with ischaemic heart disease. *Figure S1*. The Kaplan–Meier analysis of all-cause death with or without myocardial infarction. *Figure S2*. The Kaplan–Meier analysis for all-cause death with or without ventricular arrhythmia. *Figure S3*. The Kaplan–Meier analysis of all-cause death with or without intracranial haemorrhage.

## References

[1] Iqbal J., Zhang Y.-J., Holmes D. R. (2015). Optimal medical therapy improves clinical outcomes in patients undergoing revascularization with percutaneous coronary intervention or coronary artery bypass grafting. *Circulation*.

[2] Boden W. E., O’Rourke R. A., Teo K. K. (2007). Optimal medical therapy with or without PCI for stable coronary disease. *New England Journal of Medicine*.

[3] Windecker S., Kolh P., Alfonso F. (2014). 2014 ESC/EACTS guidelines on myocardial revascularization: the task force on myocardial revascularization of the European society of cardiology (ESC) and the European association for cardio-thoracic surgery (EACTS) developed with the special contribution of the European association of percutaneous cardiovascular interventions (EAPCI). *European Heart Journal*.

[4] Fihn S. D., Gardin J. M., Abrams J. (2012). 2012 ACCF/AHA/ACP/AATS/PCNA/SCAI/STS guideline for the diagnosis and management of patients with stable ischemic heart disease: executive summary. *Circulation*.

[5] Levine G. N., Bates E. R., Blankenship J. C. (2011). 2011 ACCF/AHA/SCAI guideline for percutaneous coronary intervention: a report of the American college of cardiology foundation/American heart association task force on practice guidelines and the society for cardiovascular angiography and interventions. *Circulation*.

[6] Thygesen K., Alpert J. S., Jaffe A. S. (2012). Third universal definition of myocardial infarction. *Circulation*.

[7] Chesebro J. H., Knatterud G., Roberts R. (1987). Thrombolysis in myocardial infarction (TIMI) trial, phase I: a comparison between intravenous tissue plasminogen activator and intravenous streptokinase. clinical findings through hospital discharge. *Circulation*.

[8] JCS Joint Working Group (2013). Guidelines for secondary prevention of myocardial infarction (JCS 2011). *Circulation Journal*.

[9] Baigent C., Baigent C., Blackwell L. (2010). Efficacy and safety of more intensive lowering of LDL cholesterol: a meta-analysis of data from 170,000 participants in 26 randomised trials. *Lancet*.

[10] Sabatine M. S., Giugliano R. P., Keech A. C. (2017). Evolocumab and clinical outcomes in patients with cardiovascular disease. *New England Journal of Medicine*.

[11] Bohula E. A., Giugliano R. P., Cannon C. P. (2015). Achievement of dual low-density lipoprotein cholesterol and high-sensitivity C-reactive protein targets more frequent with the addition of ezetimibe to simvastatin and associated with better outcomes in IMPROVE-IT. *Circulation*.

[12] Yamashita Y., Shiomi H., Morimoto T. (2017). Cardiac and noncardiac causes of long-term mortality in ST-segment-elevation acute myocardial infarction patients who underwent primary percutaneous coronary intervention. *Circulation: Cardiovascular Quality and Outcomes*.

[13] Winkel P., Jakobsen J. C., Hilden J. (2018). Prognostic value of routinely available data in patients with stable coronary heart disease. A 10-year follow-up of patients sampled at random times during their disease course. *Open Heart*.

[14] Inohara T., Kohsaka S., Miyata H. (2017). Prognostic impact of subsequent acute coronary syndrome and unplanned revascularization on long-term mortality after an index percutaneous coronary intervention: a report from a Japanese multicenter registry. *Journal of the American Heart Association*.

[15] Kereiakes D. J., Yeh R. W., Massaro J. M. (2016). DAPT score utility for risk prediction in patients with or without previous myocardial infarction. *Journal of the American College of Cardiology*.

[16] Rosa S. D., Sabatino J., Polimeni A., Sorrentino S., Indolfi C. (2020). Dual anti-thrombotic treatment with direct anticoagulants improves clinical outcomes in patients with atrial fibrillation with ACS or undergoing PCI. A systematic review and meta-analysis. *PLoS One*.

[17] Valgimigli M., Bueno H., Byrne R. A. (2018). 2017 ESC focused update on dual antiplatelet therapy in coronary artery disease developed in collaboration with EACTS: the task force for dual antiplatelet therapy in coronary artery disease of the European society of cardiology (ESC) and of the European association for cardio-thoracic surgery (EACTS). *European Heart Journal*.

