# Supplementary material for: Major Bleeding Events Are Stronger Predictors of Long-Term Mortality Than Coronary Events in Secondary Prevention Therapy for Ischaemic Heart Disease
Source: J Interv Cardiol. 2020 Nov 30;2020:9303750. doi: 10.1155/2020/9303750 (PMC7721507; doi:10.1155/2020/9303750)
Supplement: Supplementary Materials — Table S1. Current morbidity, risk factor control, and medication for patients with ischaemic heart disease. Figure S1. The Kaplan–Meier analysis of all-cause death with or without myocardial infarction. Figure S2. The Kaplan–Meier analysis for all-cause death with or without ventricular arrhythmia. Figure S3. The Kaplan–Meier analysis of all-cause death with or without intracranial haemorrhage. [file 9303750.f1.pdf]

Supplementary Materials

Table S1. Current morbidity, risk factor control, and medication for patients with ischaemic heart disease

**Risk Factor, %**		
Hypertension		93.0%
Diabetes mellitus		36.1%
Dyslipidaemia		95.4%
**Risk factor control**		
BP, mmHg	Systolic	133.7 ± 16.5
	Diastolic	75.0 ± 11.8
HbA1c in DM patients, %		7.0 ± 1.1
LDL-C, mg/dL		88.0 ± 21.3
**Medication, %**		
Antiplatelet therapy		
Aspirin		82.0%
Thienopyridine		26.3%
Cilostazol		1.9%
Single		40.4%
Dual		52.3%
Triple		0.1%
OAC		
VKA		8.3%
DOAC		3.4%
SAPT + OAC		8.9%
DAPT + OAC		0.9%
Statin		90.0%
EPA		6.0%
ACEI, ARB		63.0%
Beta-blocker		27.0%
CCB		66.7%

Abbreviations: BP, blood pressure; HbA1c, glycated haemoglobin; LDL-C, low-density lipoprotein cholesterol; OAC, oral anticoagulation; VKA, vitamin K antagonist; DOAC, direct oral anticoagulant;

SAPT, single antiplatelet therapy; DAPT, dual antiplatelet therapy; EPA, eicosapentaenoic acid; ACEI, angiotensin-converting enzyme inhibitor; ARB, angiotensin receptor blocker; CCB, calcium channel blocker.

Figure S1. The Kaplan–Meier analysis for all cause death with or without myocardial infarction

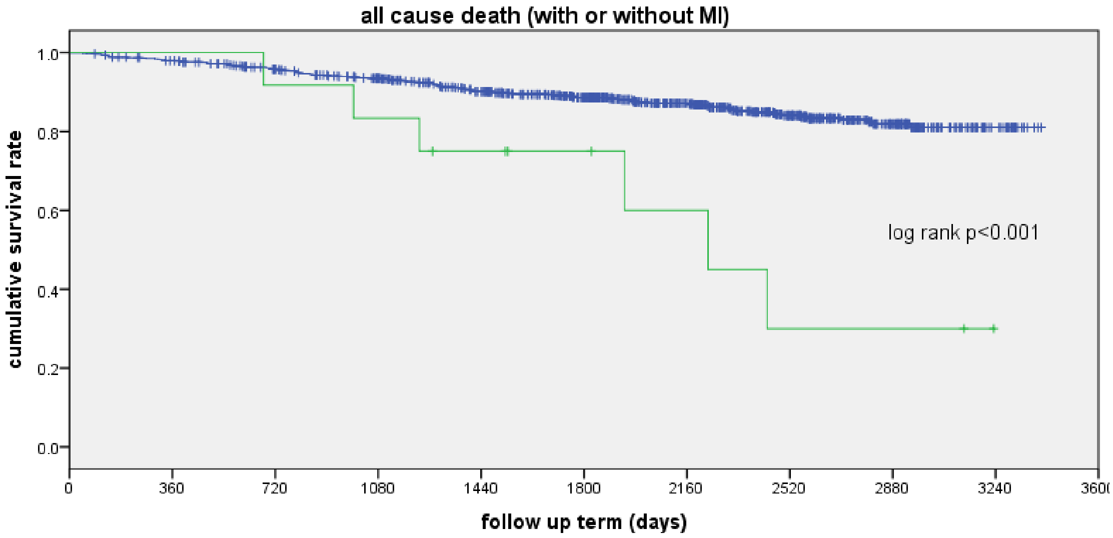

Abbreviations: MI, myocardial infarction.

Figure S2. The Kaplan–Meier analysis for all cause death with or without ventricular arrhythmia

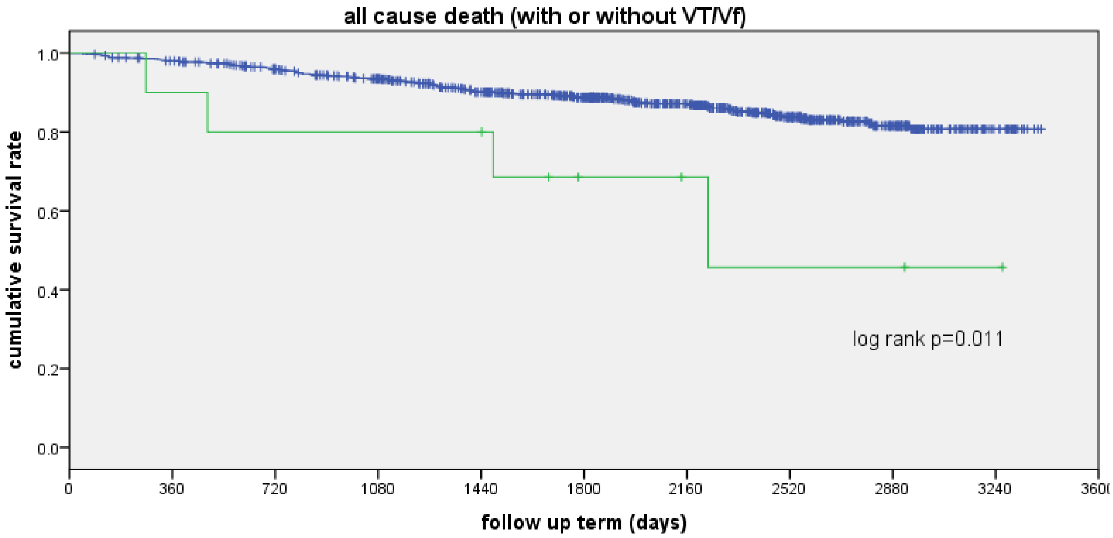

Abbreviations: VT, ventricular arrhythmia; Vf, ventricular fibrillation.

Figure S3. The Kaplan–Meier analysis for all cause death with or without intracranial haemorrhage

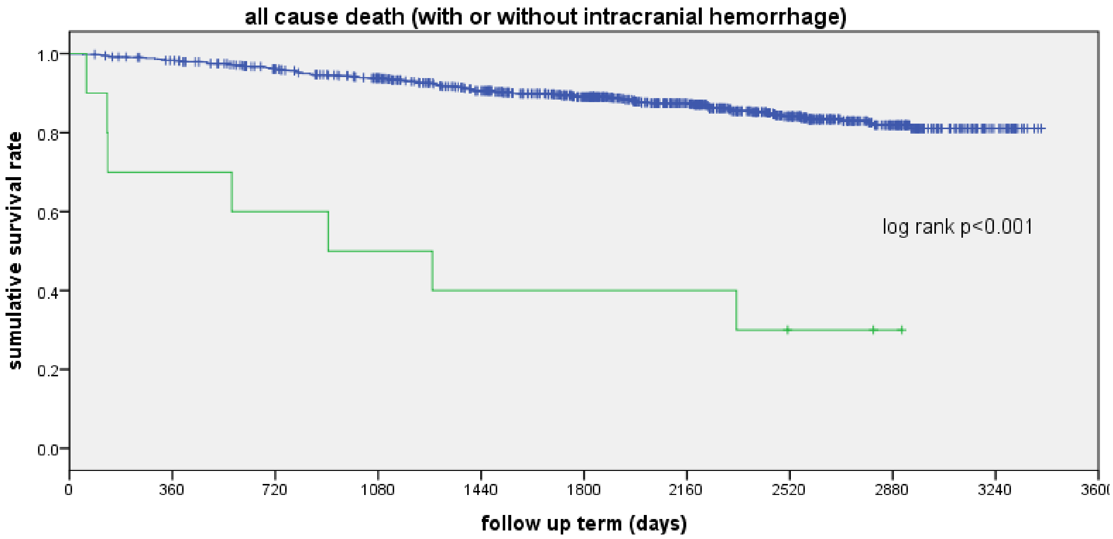
all cause death (with or without intracranial hemorrhage)
sumulative survival rate
1.0
0.8
0.6
0.4
0.2
0.0
log rank p<0.001
0
360
720
1080
1440
1800
2160
2520
2880
3240
3600
follow up term (days)
